# A Simulation Study to Assess Indicators of Antimicrobial Use as Predictors of Resistance: Does It Matter Which Indicator Is Used?

**DOI:** 10.1371/journal.pone.0145761

**Published:** 2015-12-23

**Authors:** Elise Fortin, Caroline Quach, Patricia S. Fontela, David L. Buckeridge, Robert W. Platt

**Affiliations:** 1 Department of Epidemiology, Biostatistics and Occupational Health, McGill University, Montréal, Québec, Canada; 2 Direction des risques biologiques et de la santé au travail, Institut national de santé publique du Québec, Québec and Montréal, Québec, Canada; 3 Department of Pediatrics, The Montréal Children's Hospital, McGill University, Montréal, Québec, Canada; Ross University School of Veterinary Medicine, SAINT KITTS AND NEVIS

## Abstract

**Objective:**

Indicators of antimicrobial use have been described previously, but few studies have compared their accuracy in prediction of antimicrobial resistance in hospital settings. This study aimed to identify conditions under which significant differences would be observed in the predictive accuracy of indicators in the context of surveillance of intensive care units (ICUs).

**Methods:**

Ten resistance / antimicrobial use combinations were studied. We used simulation to determine if Québec’s network of 81 ICUs or the National Healthcare Safety Network (NHSN) of 2952 ICUs are large enough to allow the detection of predetermined differences between the most accurate and 1) the second most accurate indicator, and 2) the least accurate indicator, in more than 80% of simulations. For each indicator, we simulated absolute errors in prediction for each ICU and each 4-week period, for surveillance lasting up to 5 years. Absolute errors were generated following a binomial distribution, using mean absolute errors (MAEs) observed in 9 ICUs as the average proportion; simulated MAEs were compared using t-tests. This was repeated 1000 times per scenario.

**Results:**

When comparing the two most accurate indicators, 80% power was reached less often with the Québec network versus the NHSN (0/20 versus 2/20 scenarios, with 5 years of surveillance data), a finding reinforced when comparing the most and least accurate indicators (3/20 versus 20/20 scenarios). When simulating 1 year of data, scenarios reaching an 80% power dropped to 0/20, comparing the two most accurate indicators with the larger network, and to 1/20, comparing the most and least accurate indicators with the smaller network.

**Conclusion:**

Most of the time (72%), identifying an indicator of antimicrobial use predicting antimicrobial resistance with a better accuracy was not possible. The choice of an indicator for an eventual surveillance system should rely on criteria other that predictive accuracy.

## Introduction

Surveillance of both antimicrobial resistance and population antimicrobial use are necessary to understand the magnitude of resistance problems in hospitals and obtain data for the development of tailored interventions. In Canada, surveillance of selected resistant microorganisms is already ongoing but surveillance of hospital antimicrobial use is very limited.[1–3] The Québec Ministry of Health has thus recommended the development of local surveillance in Québec healthcare facilities.[4] The optimal way to measure antimicrobial use in hospital populations, to complete surveillance of resistance, is however unclear and has been the object of long lasting debates.[5–9]

The World Health Organization recommends the use of defined daily doses per patient-days, the American National Healthcare Safety Network prefers days of treatment (agent-days) per patient-days, while the European Surveillance of Antimicrobial Consumption also measures hospital antimicrobial use with point prevalence surveys (proportion of patients receiving treatment).[10–12] A variety of indicators have also been used, such as grams per patient-days, currency per patient-days, recommended daily doses in mg/kg per patient-days, exposed patients / admissions, agent-days / admissions.[8, 13–16] Various sets of indicators have been suggested.[6, 17–19] Although some studies did compare a few indicators, very few studies compared their ability to predict levels of antimicrobial resistance in hospitals. We conducted a systematic literature review aiming to identify such studies, as long as they included pediatric populations and we found only one study comparing indicators’ correlation with resistance.[20] This study compared two of the 26 different indicators reported in the literature.[21]

In a recent study comparing the accuracy of 15 indicators of antimicrobial use in predicting resistance of the respiratory microbiota (both prevalence and incidence of resistance), no indicator was clearly superior to the others.[22] However, only nine intensive care units (ICUs) participated in the study (4 adult ICUs, 2 pediatric ICUs and 3 neonatal ICUs), raising the question of a potential lack of power to discriminate between the accuracy of indicators. This simulation study aimed to determine under which conditions significant differences would be observed among indicators in the predictive accuracy of antimicrobial resistance. We aimed to determine if, given previously observed non-statistically significant differences between indicators in absolute errors, differences could be detected in two simulated larger networks of ICUs. Our secondary objective was to evaluate the impact of follow-up duration on our results.

## Methods

This study was approved by the Research Ethics Boards of McGill University and of the *Centre Hospitalier Universitaire* Sainte-Justine. No consent from patients was necessary as the data was analyzed anonymously.

### Variables

#### Resistance / antimicrobial use combinations

Ten resistance / antimicrobial use combinations (combinations) were studied: 1) methicillin-resistant *Staphylococcus aureus* (MRSA) / penicillin use; 2) MRSA / penicillin, third-generation cephalosporins (3GC) and quinolone use; 3) piperacillin-tazobactam-resistant coliforms (PTRC) / piperacillin-tazobactam use; 4) quinolone-resistant coliforms (QRC) / quinolone use; 5) aminoglycoside-resistant coliforms (ARC) / aminoglycoside use; 6) carbapenem-resistant *E*. *coli*, *Klebsiella* sp. and *Proteus* sp. (CREKP) / carbapenem use; 7) CREKP / aminoglycoside, 3GC and quinolone use; 8) piperacillin-tazobactam-resistant *Pseudomonas* sp. (PTRP) / piperacillin-tazobactam use; 9) quinolone-resistant *Pseudomonas* sp. (QRP) / quinolone use and 10) carbapenem-resistant *Pseudomonas* sp. (CRP) / carbapenem use. These combinations were chosen based on their clinical relevance and on the frequency of resistance. Prevalence of resistance per admissions and incidence rates per patient-days were both studied, analyzed per ICU and per 4-week period. Penicillins, 3GC, quinolones (more precisely, fluoroquinolones), piperacillin-tazobactam, aminoglycosides and carbapenems respectively correspond to codes J01CA-E-F, J01DD, J01MA, J01CR05, J01G and J01DH, according to the Anatomical Therapeutic Chemical classification system.[10]

#### Indicators of antimicrobial use

This study focused on 5 numerators and 3 denominators previously identified in a systematic review of indicators of antimicrobial use in hospitalized patients populations that included pediatric populations.[20] Numerators were: 1) defined daily doses (DDD; one DDD is the average quantity, in grams, given to a 70 kg adult for 1 day; values are identical worldwide), 2) recommended daily doses (RDD; similar to DDD, but the standard daily doses are defined by local guidelines; accounting for pediatric patients’ weight in mg / kg), 3) agent-days (patient-days when a specific antimicrobial was prescribed), 4) courses (distinct periods of consecutive days when a patient is prescribed a specific antimicrobial) and 5) exposed patients (patients prescribed antimicrobials). Denominators were: 1) patient-days (sum of days spent in ICUs by individual patients, where admission and discharge days each counted for half a day), 2) admissions (including transfers from other wards) and 3) patients present. Fifteen indicators of use of different antimicrobial classes were thus studied, per ICU and per 4-week period.

#### Predictive accuracy

The accuracy of indicators in predicting of the prevalence of resistant respiratory microbiota organisms was measured using mean absolute errors (MAEs).[23] A MAE is a measure of accuracy used in the prediction of time series as it measures the mean difference between observed and model-predicted values; MAEs obtained with different models can be compared using t-tests. In the original cohort study, regression models were used to model prevalence and incidence rates of resistance, per ICU and per 4-week period, successively using the fifteen indicators of antimicrobial use, after adjusting for ICU type (adult, pediatric or neonatal).[22] For each combination, 60 models were built for prevalence (15 indicators x 4-week time lag or no time lag x additive or multiplicative models) and 60 others for incidence rates. MAEs were computed for each model. Errors are the observed prevalence (or observed incidence) minus prevalence (or incidence) predicted by the model. Absolute values of these errors are then averaged, to obtain the MAE. A smaller MAE indicates a more accurate model. For example, in predicting CRP prevalence with carbapenem use, the most accurate model was an additive model with carbapenem use measured in courses per 100 patient-days. With no time lag, this model had a MAE of 0.31 cases per 100 admissions (0.46 for adult ICUs, 0.26 for pediatric ICUs and 0.15 for neonatal ICUs). The second most accurate model was also additive, used no time lag and used carbapenem use measured in agent-days per 100 patient-days for a MAE of 0.32 cases per 100 admissions (0.48 for adult ICUs, 0.24 for pediatric ICUs and 0.14 for neonatal ICUs). Finally, the least accurate model was multiplicative, had a MAE of 0.43 cases per 100 admissions (0.50 for adult ICUs, 0.20 for pediatric ICUs and 0.50 for neonatal ICUs) and used carbapenem use measured in recommended daily doses per 100 admissions, with a 4-week-period time lag. S1 Fig illustrates this example.

### Simulation procedures

Forty scenarios were studied for each combination (Table 1): 1) for the prediction of prevalence, ten scenarios where the most accurate indicator was compared to the second most accurate indicator (two networks of ICUs x five different durations of surveillance) and ten scenarios where the most accurate indicator was compared to the least accurate indicator; 2) the same twenty scenarios were also simulated for the prediction of incidence rates. One thousand independent simulations were performed per scenario. For each simulation run, the same seed was used to produce the absolute errors for the two indicators to be compared (but with different mean absolute errors) because the original study compared MAEs obtained while trying to predict the same outcome and were thus dependent. As a result, compared indicators were simulated using the same seed, but each scenario’s 1000 simulations were independent. Indicators were compared using the Satterthwaite t-test method, as we could not assume that compared MAEs would always have equal variances. Simulations were performed using SAS 9.3; datasets were created in data steps, creating random binomial variables using call ranbin routines.

**Table 1:** Scenarios studied to assess power to detect differences between indicators in predicting prevalence and incidence rates of resistance (1000 simulations per scenario). Note: 3GC: third-generation cephalosporins; amino: aminoglycosides; ARC: aminoglycoside-resistant coliforms; CREKP: carbapenem-resistant *E*. *coli*, *Klebsiella* sp. and *Proteus* sp.; CRP: carbapenem-resistant *Pseudomonas* sp.; ICU: intensive care unit; MRSA: methicillin-resistant *Staphylococcus aureus*; NHSN: National Healthcare Security Network; pip-tazo: piperacillin-tazobactam; PTRC: piperacillin-tazobactam-resistant coliforms; PTRP: piperacillin-tazobactam-resistant *Pseudomonas* sp.; QRC: quinolone-resistant coliforms; QRP: quinolone-resistant *Pseudomonas* sp.; SPIN-BACTOT: Québec healthcare-associated bloodstream infections network.

Measure of resistance	Resistance	Antimicrobial use	SPIN-BACTOT network (Most accurate indicator vs…)		NHSN (Most accurate indicator vs…)	
			Second most accurate	Least accurate	Second most accurate	Least accurate
Prevalence (/admissions)	MRSA	Penicillins	1, 2, 3, 4 and 5 years of data	1, 2, 3, 4 and 5 years of data	1, 2, 3, 4 and 5 years of data	1, 2, 3, 4 and 5 years of data
	MRSA	Penicillins + 3GC + quinolones				
	PTRC	Piperacillin-tazobactam				
	QRC	Quinolones				
	ARC	Aminoglycosides				
	CREKP	Carbapenems				
	CREKP	Aminoglycosides + 3GC + quinolones				
	PTRP	Piperacillin-tazobactam				
	QRP	Quinolones				
	CRP	Carbapenems				
Incidence rate (/patient-days)	MRSA	Penicillins	1, 2, 3, 4 and 5 years of data	1, 2, 3, 4 and 5 years of data	1, 2, 3, 4 and 5 years of data	1, 2, 3, 4 and 5 years of data
	MRSA	Penicillins + 3GC + quinolones				
	PTRC	Piperacillin-tazobactam				
	QRC	Quinolones				
	ARC	Aminoglycosides				
	CREKP	Carbapenems				
	CREKP	Aminoglycosides + 3GC + quinolones				
	PTRP	Piperacillin-tazobactam				
	QRP	Quinolones				
	CRP	Carbapenems				

For each scenario, we generated datasets containing the absolute errors for each of the indicators of antimicrobial use compared, per ICU and per 4-week period of surveillance. For scenarios investigating the prediction of resistance prevalence, absolute errors represented differences between two proportions (observed–predicted). Absolute error per 4-week period = *x* / average number of admissions per 4-week period, where X ~ Bin (average number of admissions per 4-week period, observed MAE). For scenarios investigating the prediction of resistance incidence rates, absolute errors represented differences between two rates and number of admissions was replaced by number of patient-days. As observed MAEs varied according to ICU type, random variables were generated stratifying per ICU type.

Patient-days and admissions per type of ICU (pediatric, neonatal and adult) followed the structure of two existing networks of ICUs: the Québec healthcare-associated bloodstream infections surveillance network (SPIN-BACTOT, 2009–2010) and the American National Healthcare Security Network (NHSN, 2009).[24, 25] Characteristics of these networks are summarized in Table 2. Patient-days were available for both SPIN-BACTOT and NHSN ICUs, but admissions were unknown. The average number of patient-days per period was computed. From data observed in the nine ICUs participating to the original cohort study, we computed the ratio of admissions per patient-day, per ICU type (0.21 for adult ICUs, 0.21 for pediatric ICU and 0.07 for neonatal ICUs). We then estimated the average periodic number of admissions in SPIN-BACTOT and NHSN by multiplying this ratio by the number of patient-days reported in each network. Simulations were run for surveillance durations ranging from 13 to 65 periods 4-week periods (from 1 to 5 years).

**Table 2:** Description of the SPIN-BACTOT and NHSN networks. Note: ICU: intensive care unit; NHSN: National Healthcare Security Network; SPIN-BACTOT: Québec healthcare-associated bloodstream infections network.

ICU type	SPIN-BACTOT			NHSN		
	ICU (N)	Patient-days (N, /4-week period and / ICU)	Admissions (N, estimated, /4-week period and / ICU)	ICU (N)	Patient-days (N, /4-week period and / ICU)	Admissions (N, estimated, /4-week period and / ICU)
Adult	70	199	42	2591	255	54
Pediatric	4	120	25	178	253	52
Neonatal	7	514	36	183	867	61

For each simulation, a t-statistic comparing the smallest MAE to the other MAEs was computed and p-values stored. The methodology used in the initial cohort study presumed that all indicators were compared to the most accurate one: all 60 models of a given scenario were ranked according to their MAE; if the least accurate model was not statistically different from the most accurate one, then all other models were assumed to not be different. A Holm correction was thus applied to account for multiple comparisons. For scenarios comparing the two most accurate indicators, when 80% of simulations had a p-value below 0.05 (0.05 / 1), we considered that this scenario had an 80% power to detect a difference between the two indicators compared. The significance level was rather 0.0008 (0.05 / 59) when comparing the most and the least accurate indicators.

## Results

### Accuracy in the prediction of resistance prevalence

**Fig 1:**
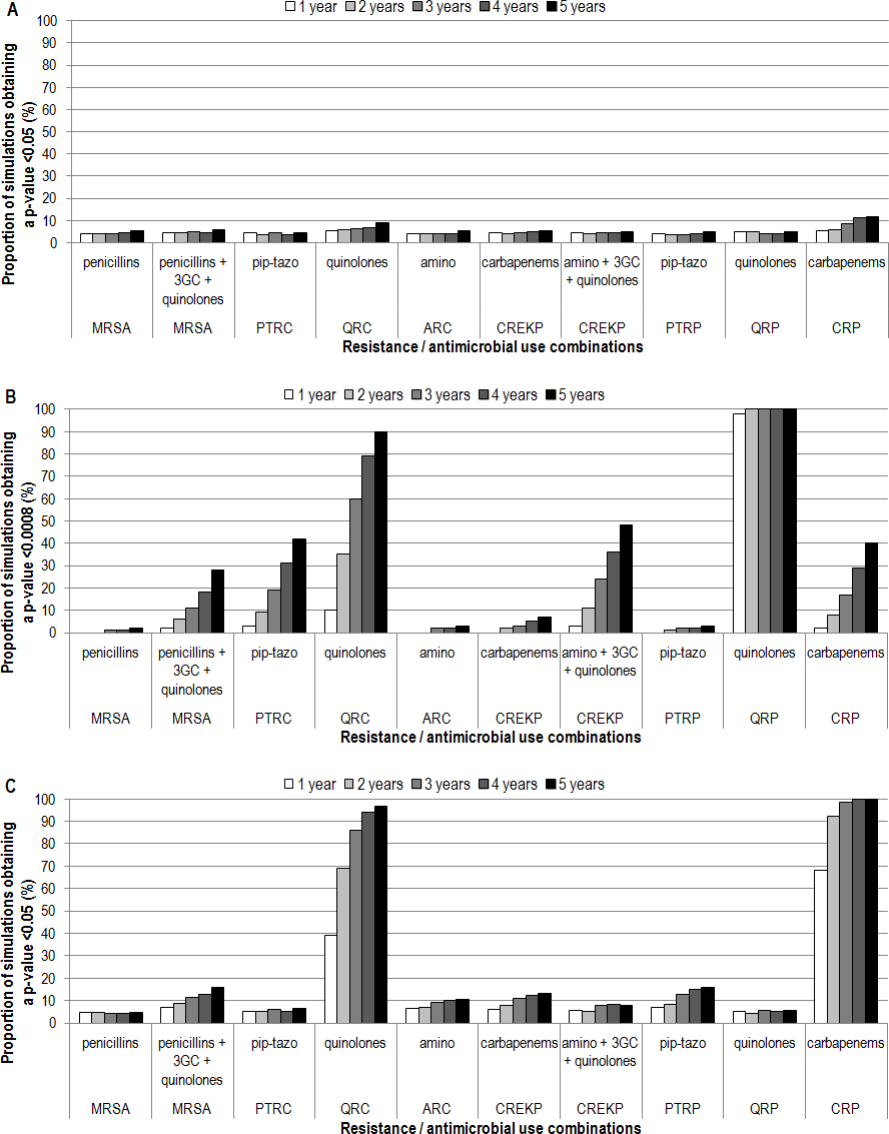
Proportion of simulations detecting differences between indicators in predicting resistance prevalence, for ten combinations and five durations. A) Network of ICUs similar to SPIN-BACTOT’s ICU network, comparing the two most accurate indicators. B) Network of ICUs similar to SPIN-BACTOT’s ICU network, comparing the most accurate indicator to the least accurate. C) Network of ICUs similar to the NHSN, comparing the two most accurate indicators. 3GC: third-generation cephalosporins; amino: aminoglycosides; ARC: aminoglycoside-resistant coliforms; CREKP: carbapenem-resistant *E*. *coli*, *Klebsiella* sp. and *Proteus* sp.; CRP: carbapenem-resistant *Pseudomonas* sp.; ICU: intensive care unit; MRSA: methicillin-resistant *Staphylococcus aureus*; NHSN: National Healthcare Security Network; pip-tazo: piperacillin-tazobactam; PTRC: piperacillin-tazobactam-resistant coliforms; PTRP: piperacillin-tazobactam-resistant *Pseudomonas* sp.; QRC: quinolone-resistant coliforms; QRP: quinolone-resistant *Pseudomonas* sp.; SPIN-BACTOT: Québec healthcare-associated bloodstream infections network.

Using a network of ICUs similar to SPIN-BACTOT’s ICU network (70 adult ICUs, 4 pediatric ICUs and 7 neonatal ICUs), we were unable to distinguish the best of the two most accurate indicators, regardless of surveillance duration (Fig 1A). Differences could be found between the most and the least accurate indicators in 80% of simulations for two combinations (Fig 1B). These differences could only be detected after 5 years of surveillance for QRC / quinolone use. For QRP / quinolone use, a difference was observed even after only 1 year of surveillance.

With a network of ICUs similar to NHSN (2591 adult ICUs, 178 pediatric ICUs and 183 neonatal ICUs), the two most accurate indicators could only be distinguished for two combinations (Fig 1C). For CRP / carbapenem use, 2 years of surveillance were sufficient while for QRC / quinolone use, 3 years were necessary. Differences could always be found between the most and the least accurate indicators, for all 10 combinations except MRSA / penicillin use, for which at least 2 years of data were necessary.

### Accuracy in the prediction of resistance incidence rates

**Fig 2:**
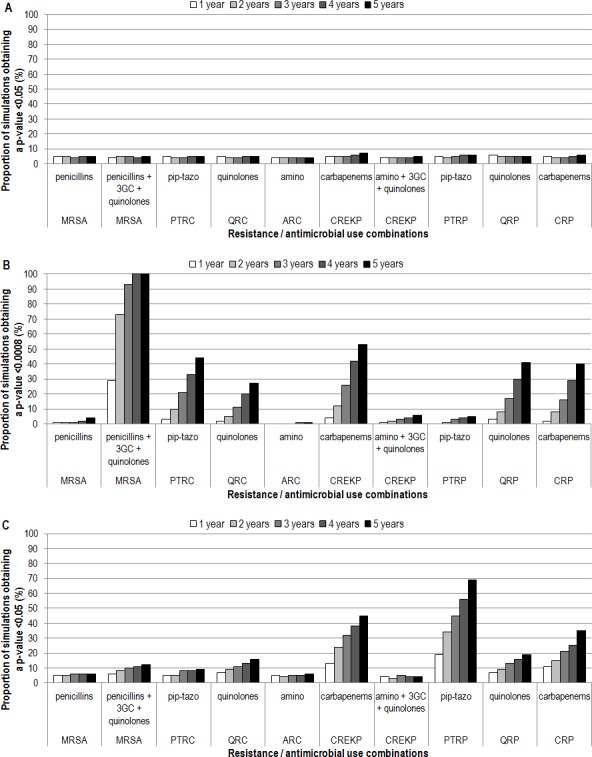
Proportion of simulations detecting differences between indicators in predicting resistance incidence rates, for ten combinations and five durations. A) Network of ICUs similar to SPIN-BACTOT’s ICU network, comparing the two most accurate indicators. B) Network of ICUs similar to SPIN-BACTOT’s ICU network, comparing the most accurate indicator to the least accurate. C) Network of ICUs similar to the NHSN, comparing the two most accurate indicators. 3GC: third-generation cephalosporins; amino: aminoglycosides; ARC: aminoglycoside-resistant coliforms; CREKP: carbapenem-resistant *E*. *coli*, *Klebsiella* sp. and *Proteus* sp.; CRP: carbapenem-resistant *Pseudomonas* sp.; ICU: intensive care unit; MRSA: methicillin-resistant *Staphylococcus aureus*; NHSN: National Healthcare Security Network; pip-tazo: piperacillin-tazobactam; PTRC: piperacillin-tazobactam-resistant coliforms; PTRP: piperacillin-tazobactam-resistant *Pseudomonas* sp.; QRC: quinolone-resistant coliforms; QRP: quinolone-resistant *Pseudomonas* sp.; SPIN-BACTOT: Québec healthcare-associated bloodstream infections network.

With a network of ICUs similar to SPIN-BACTOT’s ICU network, the two most accurate indicators could never be distinguished (Fig 2A). Also, 80% power could be reached for only 1 of 10 scenarios in the detection of differences between the most and the least accurate indicators (MRSA / penicillin, 3GC and quinolone use), and it necessitated 3 years of surveillance data. (Fig 2B).

With a network of ICUs similar to NHSN, even though more simulations detected differences, 80% power was never reached when comparing the two most accurate indicators (Fig 2C). Differences could always be found between the most and the least accurate indicators, for all 10 combinations, however, 3 years of data were necessary for ARC / aminoglycoside use.

## Discussion

This simulation study has allowed us to compare predictive accuracy of different indicators of antimicrobial use, while exploring conditions for which a specific indicator should be selected among others, to improve surveillance. This study was for surveillance purposes. Surveillance of both AM use and resistance are recommended by public health instances not to demonstrate the existence between the two variables (which is widely accepted) nor to quantify it, but to use it for hypothesis generation and to evaluate the impact of interventions on inpatient populations. We estimated the power necessary to distinguish indicators of antimicrobial use regarding their accuracy in predicting antimicrobial resistance in networks of ICUs. Networks of ICUs were simulated, similar in size and structure to a provincial network (SPIN-BACTOT) and to a much larger network (NHSN). Absolute errors were simulated for each ICU, per 4-week period and mean absolute errors were compared. Results of this study show us that network size and surveillance duration influence power to detect differences between MAEs, but that most of the time, MAEs (i.e. indicators of antimicrobial use) showed similar predictive accuracies.

The size of ICU networks had an important impact on our ability to distinguish indicators of antimicrobial use. Indeed, when comparing the two most accurate indicators, 80% power was reached less often with the Québec network versus the NHSN (0 / 20 scenarios versus 2 / 20 scenarios, respectively, with 5 years of surveillance data). This was especially true when comparing the most and least accurate indicators (3 / 20 scenarios versus 20 / 20 scenarios, respectively). In the scenarios less likely to detect differences between MAEs (comparing the two most accurate indicators in the provincial network), duration of surveillance did not influence the capacity to reach 80% power: such a network was underpowered to detect differences, even with five years of data. Similarly, duration of surveillance was irrelevant in the scenarios most likely to detect differences between MAEs (comparing the most and the least accurate indicators in the large national network), as a single year of data was usually sufficient to reach 80% power. However, the accumulation of more data through increased surveillance duration did make a difference in other scenarios: when simulating only 1 year of surveillance data, scenarios allowing to reach 80% power dropped from 2 to 0 / 20, comparing the two most accurate indicators in the larger network, and from 3 to 1 / 20, comparing the most and the least accurate indicators in the smaller network.

In a systematic review of indicators of antimicrobial use in hospitalized patients populations that included pediatric populations, 26 indicators were identified, combining 13 numerators and 5 denominators.[20] Some numerators identified in the systematic review were not kept for the simulations: information provided by grams and costs is reflected in DDDs and RDDs, but blurred through market fluctuations; prescribed daily doses and agent-days should be equivalent, so only agent-days were kept; as patients’ weights are not always known (especially in adults), RDDs and RDD in mg/kg were combined into a single measure. Finally, as we stratified our indicators per antimicrobial class, antimicrobial-days and treatment periods became almost identical to agent-days and courses (respectively) to warrant additional analyses. Regarding denominators, costs and kg-days, also identified in the systematic review, were not kept in the analyses because, once again, market fluctuations also limit the use of costs and patients’ weights are not always known.

Interpretation of results is limited by assumptions made in the simulation procedures. First, we assumed that the ideal design was to predict resistance at the ICU level rather than pooled provincial or national resistance prevalence or incidence rate. We also assumed that surveillance would be performed on a 4-week or monthly basis rather than on an annual basis, to follow time variations. In this setting, the larger the number of participating ICUs and the finer the time intervals, the more observations are produced, increasing power to detect differences between indicators. Even if a surveillance system was to eventually pool all data in a single annual estimate of resistance, in a project like ours, trying to identify the indicator that predicts resistance levels with the best accuracy, finer observation units allowed us to reduce a potential ecological bias. Second, we assumed that values observed in the initial cohort study (admissions: patient-days ratios and MAEs) are representative of entire networks of ICUs; we also assumed that ICU type (adult, pediatric and neonatal ICUs) is the only relevant element in the structure of ICU networks. As the number of studies comparing predictive accuracy of indicators of population antimicrobial use is quite small, we performed this simulation study using available information (MAEs we already had). Third, available information for our simulations related to ICUs, rather than hospitals. Length of stay is longer when considering the entire hospital and antimicrobial use varies between wards.[8] Although this simulation study is certainly a first hint on the population size necessary to identify a more accurate indicator, results might differ at the hospital level. Similar studies at hospital level would be an interesting complement to our findings. Finally, as statistically significant differences were not observed in the initial cohort study, the present simulation study could not identify the most accurate indicator (or indicators, as they could vary between combinations); our study was only designed to estimate power that could be reached with different ICU networks sizes and surveillance durations, to eventually identify the most accurate indicator of antimicrobial use. However, we believe that the lack of evidence of differences reflects absence of differences, rather than being inconclusive.

## Conclusion

Network size and surveillance duration influence power to detect differences between indicators. However, most of the time, identifying an indicator of antimicrobial use predicting antimicrobial resistance with a better accuracy was not possible. The choice of an indicator for an eventual surveillance system could rely on criteria other than predictive accuracy, such as feasibility (ease of data collection and computation) and the potential for external comparisons, without decreasing the quality of their surveillance activities. Results also confirm that the incapacity to observe statistically significant differences in this previous study was not due to a blatant lack of statistical power. Ideally, both the cohort and the simulation studies should be reproduced, using other surveillance conditions in confirmatory studies. Our studies are however a first answer to a long existing question; they also propose a methodological framework for future studies on this topic.

## Supporting Information

S1 Fig Methodology followed to identify the most accurate, the second most accurate and the least accurate indicators, in predicting prevalence of carbapenem-resistant *Pseudomonas* sp. in nine intensive care units. DDD: defined daily doses; ICU: intensive care unit; MAE: mean absolute error; RDD: recommended daily doses.
(TIF)

## Abbreviations

3GC: third-generation cephalosporins
Amino: aminoglycosides
ARC: aminoglycoside-resistant coliforms
CREKP: carbapenem-resistant *E*. *coli*, *Klebsiella* sp. and *Proteus* sp
CRP: carbapenem-resistant *Pseudomonas* sp
ICU: intensive care unit
MAE: mean absolute error
MRSA: methicillin-resistant *Staphylococcus aureus*
NHSN: National Healthcare Security Network
Pip-tazo: piperacillin-tazobactam
PTRC: piperacillin-tazobactam-resistant coliforms
PTRP: piperacillin-tazobactam-resistant *Pseudomonas* sp
QRC: quinolone-resistant coliforms
QRP: quinolone-resistant *Pseudomonas* sp
SPIN-BACTOT: Québec healthcare-associated bloodstream infections surveillance network
