# Supplementary figures and images for: A Simulation Study to Assess Indicators of Antimicrobial Use as Predictors of Resistance: Does It Matter Which Indicator Is Used?
Source: PLoS One. 2015 Dec 23;10(12):e0145761. doi: 10.1371/journal.pone.0145761 (PMC4689584; doi:10.1371/journal.pone.0145761)

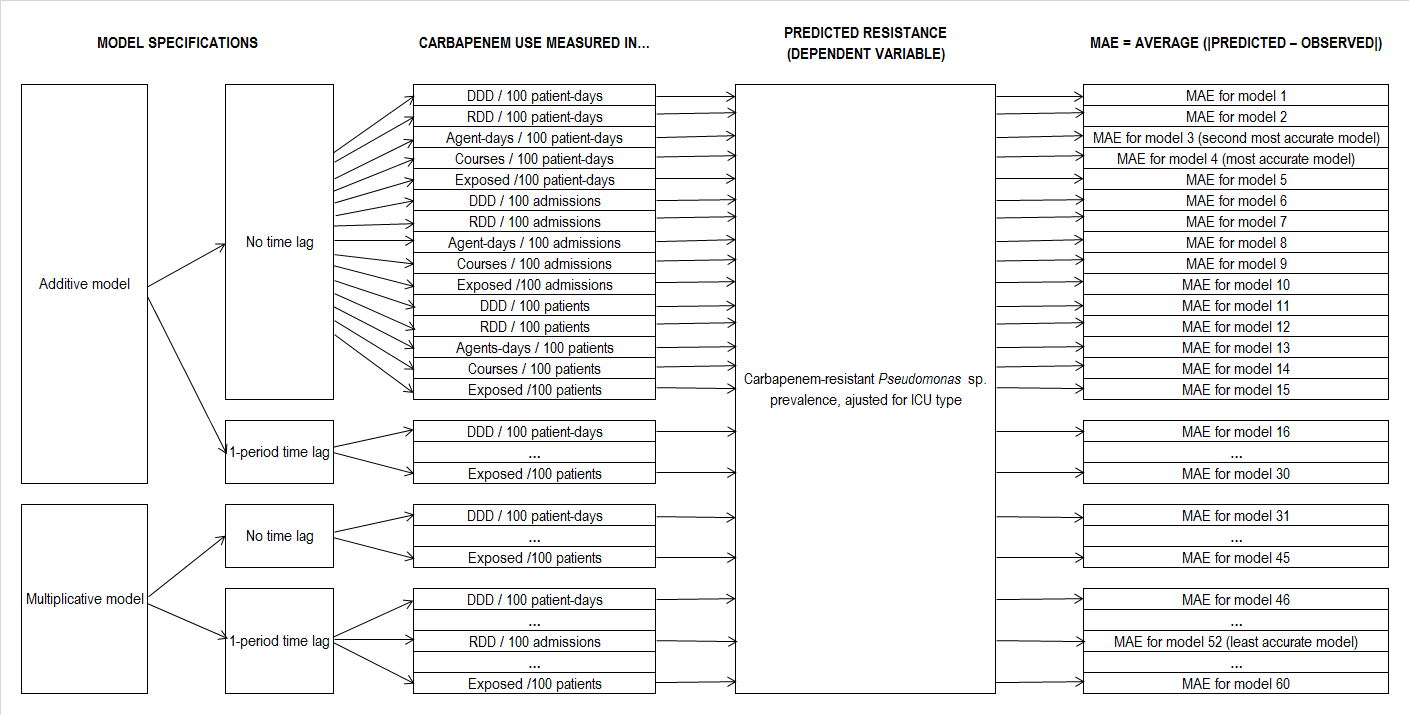

Supplement: S1 Fig — DDD: defined daily doses; ICU: intensive care unit; MAE: mean absolute error; RDD: recommended daily doses. (TIF) [file pone.0145761.s001.tif]
